# Effects of Simulated Microgravity on Ultrastructure and Apoptosis of Choroidal Vascular Endothelial Cells

**DOI:** 10.3389/fphys.2020.577325

**Published:** 2021-01-18

**Authors:** Hongwei Zhao, Yuanyuan Shi, Changyu Qiu, Jun Zhao, Yubo Gong, Chuang Nie, Bin Wu, Yanyan Yang, Fei Wang, Ling Luo

**Affiliations:** ^1^Department of Ophthalmology, The PLA Strategic Support Force Characteristic Medical Center, Beijing, China; ^2^China Astronaut Research and Training Center, Beijing, China

**Keywords:** ultrastructure, apoptosis, RCCS, CVECs, simulated microgravity

## Abstract

**Background:**

It was confirmed that simulated microgravity (SMG) led to ultrastructural alterations and apoptosis in many types of microvascular endothelial cells. However, whether SMG would also affect choroidal vascular endothelial cells (CVECs) remains unknown. This study was designed to investigate the effects of SMG on ultrastructure and apoptosis of CVECs.

**Methods:**

The rotary cell culture system (RCCS) was utilized to simulate microgravity condition. Human CVECs were cultured under normal gravity (NG) or SMG condition for 3 days. The ultrastructure was viewed under transmission electron microscopy, and the organization of F-actin was observed by immunofluorescence staining. Additionally, the apoptosis percentage was calculated using flow cytometry. Moreover, the mRNA and protein expression of BAX, Bcl-2, Caspase3, Cytochrome C, p-AKT, and p-PI3K were detected with quantitative PCR and Western blot at different exposure time.

**Results:**

In the SMG group, CVECs presented with a shrunk cell body, chromatin condensation and margination, mitochondria vacuolization, and apoptotic bodies. The amount of F-actin decreased, and the filaments of F-actin were sparse or even partly discontinuous after cultivation under SMG for 72 h. The proportions of apoptotic CVECs in SMG groups at 24 and 72 h were significantly higher than those in the NG group (*P* < 0.001). The mRNA and protein expression of Bax, Caspase3, and Cytochrome C of CVECs in SMG groups at 24 and 72 h significantly increased than those of the NG group, respectively (*P* < 0.001). The alterations of p-AKT and p-PI3K protein expression possessed similar trends. On the contrary, the mRNA and protein expression of Bcl-2 in CVECs under SMG at 24 and 72 h were significantly less than that of the NG group, respectively (*P* < 0.001).

**Conclusion:**

Simulated microgravity conditions can lead the alterations of the F-actin structure and apoptosis of CVECs. The Bcl-2 apoptosis pathway and PI3K/AKT pathway may participate in the damage of CVECs caused by SMG.

## Introduction

It is well known that cardiovascular deconditioning occurs in astronauts exposed to microgravity. It might be ascribed to vascular endothelial cell (VEC) dysfunction as VECs orchestrate vessels and blood circulation, thus exerting a pivotal role on tissue homeostasis (Girn et al., 2007; Rudimov and Buravkova, 2016). VECs are sensitive to gravity variations. Many studies demonstrated significant modifications in VECs after exposure to altered gravity conditions (Infanger et al., 2006; Grimm et al., 2009; Wehland et al., 2013; Maier et al., 2015; Janmaleki et al., 2016; Li et al., 2018). It has been confirmed that simulated microgravity(SMG) conditions lead to apoptosis of VECs and influence cell proliferation, cytoskeleton organization, and growth behavior (Carlsson et al., 2003; Cotrupi et al., 2005; Morbidelli et al., 2005; Versari et al., 2007; Mariotti and Maier, 2008; Rudimov and Buravkova, 2016).

The cytoskeleton is known to be important for maintaining normal cell morphology and function. It was believed to play a vital role in adapting to external stress, including changes in gravity. It was reported that 22 s of weightlessness produced by parabolic flight maneuvers caused rearrangement of β-tubulin and altered morphology and gene expression of VECs (Grosse et al., 2012). In human umbilical vein endothelial cells (HUVECs), exposure to SMG condition for 24 h caused 65% reduction in F-actin and about 26% in β-tubulin expression (Janmaleki et al., 2016). Similarly, SMG conditions of HUVECS for 24 h also induced cytoskeleton reorganization and morphology change (Rudimov et al., 2015). Cytoskeletal disorganization induced by exposure to SMG conditions was able to trigger the activation of autophagy or mitophagy, thereafter leading to mitochondrial loss (Locatelli et al., 2020).

Simulated microgravity conditions lead to not only alteration of cell morphology but also gene expression and then trigger apoptotic signals. It was demonstrated that SMG conditions upregulated the gene expression of FAS-L, p53, and BAX and downregulated the gene expression of PCNA and Bcl-2 in porcine aortic endothelial cells (PAECs) (Morbidelli et al., 2005). SMG condition was reported to induce apoptosis of human pulmonary microvascular endothelial cells (HPMECs) by enhancing NFκB expression and activating the PI3K/Akt pathway (Kang et al., 2011; Tang et al., 2019).

Taken together, VECs may sense the altered circumstance forces brought by SMG and induce cytoskeletal alterations then activate some second messengers which proceed to result in various responses of genes and ultimately cause apoptosis.

Choroidal vascular endothelial cells (CVECs), like the other kinds of VECs, make up the inner wall of choroidal vessels in the eyes and also play pivotal roles in regulating local blood flow as well as many physiological processes, which help to maintain tissue homeostasis. Particularly, its dysfunction may be associated with choroidal thickening which may exert influence on intraocular pressure and visual function which varied during and after spaceflight (Mader et al., 2011; Shinojima et al., 2012; Zhang and Hargens, 2018; Huang et al., 2019). However, whether CVECs are likewise sensitive to SMG has not been investigated. To explore the alterations of CVEC under SMG, we exposed CVECs to SMG using the rotary cell culture system (RCCS) and found that SMG leads to alteration of the F-actin structure and apoptosis of CVECs with activation of the Bcl-2 apoptosis pathway and PI3K/AKT pathway.

## Materials and Methods

### RCCS Bioreactor

The RCCS bioreactor (Synthecon, Houston, United States) was placed in a mixture incubator filled with CO2 and air. Microcarrier beads of 0.05 g Cytodex-3 (Sigma, United States) were pretreated with 75% ethanol and washed with 0.1 mol/l PBS for three times before being added to the rotating culture vessel. Then, the CVECs were seeded to the culture vessel at a density of approximately 5 × 10^6^ cells/vessel and cells attached to the microcarrier beads. The culture medium filled the culture chamber, and all air bubbles were removed. The rotation speed was set at 12 rpm/min. The control group sample (NG group, 1 g static culture) was placed in the same incubator. The rotation time lasted for 72 h.

### Cell Culture

The human CVEC line was obtained from Haling Biotechnology Co. Ltd (HL-CELL-0126) and was cultured in special basic medium and special additives (HL-MED-0002, HL-SUP-0002 China) at 37°C; 10% fetal bovine serum (FBS), 100 mg/ml streptomycin, and 100 units/ml penicillin (Gibco, Australia) were added to the medium.

### Transmission Electron Microscopy

Choroidal vascular endothelial cells were washed with PBS and then fixed in 2.5% glutaraldehyde at 4°C for 24 h. After being dealt with 1% OsO4 for 1 h, CVECs were washed with PBS for 30 min and were dehydrated in graded ethanol series and then embedded in Epon-812. Sections (50 microns) were stained with 2% uranyl acetate solution and 1% solution of lead citrate for 30 min. At last, the ultrastructure was examined under transmission electron microscopy (TEM) (Hitachi-HT7700, Japan).

### Immunofluorescence Staining

The F-actin of CVECs was observed by immunofluorescence staining. Briefly, CVECs were fixed with 4% paraformaldehyde for 30 min and then were incubated with a 5% FBS for 30 min at 37°C. Cells continued to be incubated with anti-F-actin (Abcam ab205, Shanghai, China) antibodies overnight at 4°C and incubated with FITC-conjugated anti-IgG (Proteintech SA00003-1, Wuhan, Hubei, China) on the next day for approximately 1 h at 37°C and DAPI (Beyotime, #C1002, 1:1500) for 2 min. Images were obtained using a confocal fluorescence microscope (Leica-LCS-SP8-STED).

### Apoptosis Assay

The CVECs were harvested using trypsin (0.25%) and washed in ice-cold PBS twice and suspended in binding buffer at a concentration of 5 × 10^5^ cells/ml. Following mixture with Annexin V-PE, the CVECs were incubated at 4°C without light for 10 min. Finally, CVECs were mixed with 7-AAD and were incubated for 5 min at 4°C in the dark. Flow cytometry was applied to detect the apoptotic cells.

### Real-Time Quantitative Polymerase Chain Reaction

Total RNA of CVECs was extracted with TRIzol (Invitrogen Life Technologies, Carlsbad, United States) and were reverse transcribed with the Prime Script RT Master Mix Kit (Takara, Tokyo, Japan) followed by the product instructions. The genes were then amplified from cDNA by PCR. The primers were as follows:

Bcl-2: forward 5′-GGTGGGGTCATGTGTGTGG-3′ and reverse 5′-CGGTTCAGGTACTCAGTCATCC-3′
Bax: forward 5′-CCCGAGAGGTCTTTTTCCGAG-3′ and reverse 5′-CCAGCCCATGATGGTTCTGAT-3′
Cyto-c: forward 5′-ACCAGGCTCACATGCCCTA-3′ and reverse 5′-TTCGATGTCACGGGATGTCAT-3′
Caspase3: forward 5′-CATGGAAGCGAATCAATGGACT-3′ and reverse 5′-CTGTACCAGACCGAGATGTCA-3′
GAPDH: forward 5′-GGAGCGAGATCCCTCCAAAAT-3′ and reverse 5′-GGCTGTTGTCATACTTCTCATGG-3′.

The steps were as follows: after denaturation (5 min at 94°C), amplification (95°C 30 s, 54–58°C 1 min and 72°C 1 min), and extension (72°CC 5 min); RT-qPCR was conducted with the One Step SYBR^®^ Prime Script^®^ PLUS RT-RNA PCR Kit (TaKaRa, Dalian, China). GAPDH was used as the internal control, and the relative expression of mRNA was calculated using the 2^–ΔΔCT^ method.

### Western Blot

The CVEC proteins were lysed in RIPA buffer containing protease inhibitors. Then, the protein concentrations were detected using the BCA Protein Assay Kit (Beyotime Biotechnology, Jiangsu, China). Next, the protein sample was electrophoresed by 8% SDS-PAGE and blocked with 5% bovine serum albumin and then was incubated with the primary antibody: anti-Bcl2 (Proteintech 60718-1-lg, Wuhan, Hubei, China), anti-Bax (Proteintech50599-2-lg, Wuhan, Hubei, China), anti-Caspase 3 (Cell Signaling Technology 9661s, Danvers, MA, United States), anti-Cytochrome C (Proteintech 10993-1-AP, Wuhan, Hubei, China), anti-p-AKT (Cell Signaling Technology 4060S, Danvers, MA, United States), anti-p-PI3K (Cell Signaling Technology 4228S, Danvers, MA, United States), and anti-GAPDH (Cell Signaling Technology 5174S, Danvers, MA, United States). Then, the primary antibody was washed out for 3 times and the membranes were incubated with a horseradish peroxidase (HRP)-conjugated goat anti-rabbit IgG secondary antibody (Cell Signaling Technology 7074S/7076S, Danvers, United States) for 1.5 h at room temperature. The intensity of immune response was observed the with Image J software (1.48u, National Institutes of Health, United States).

### Statistical Analyses

The data were expressed as the mean ± SD and were analyzed using SPSS 11.5 software (SPSS Inc., Chicago, IL, United States). One-way analysis of variance followed by Student’s *t*-test was used to determine significance of difference. All experiments were performed three times, and *P* < 0.05 was considered as a statistical difference.

## Results

### Ultrastructure of the CVECs

**FIGURE 1:**
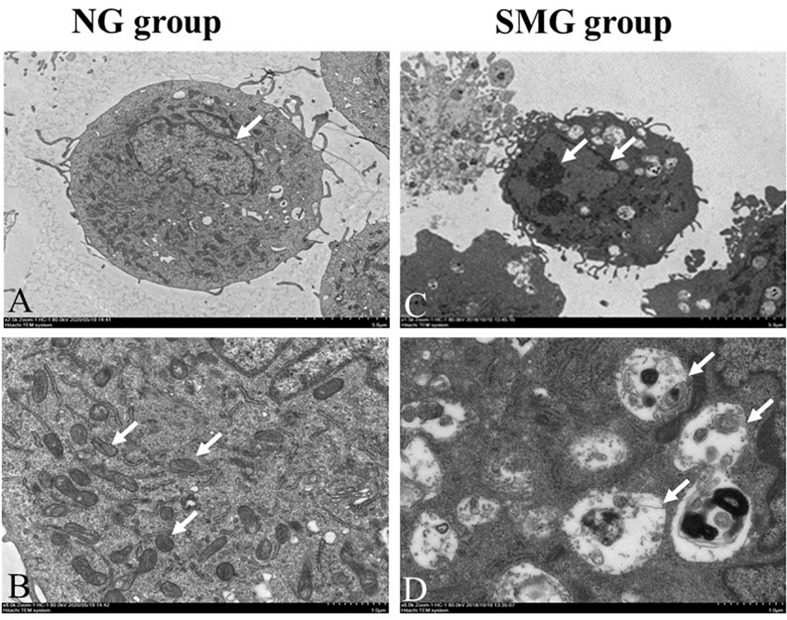
Ultrastructure of the CVECs by transmission electron microscopy. **(A,B)** Cells in the NG group. The cells presented with complete nuclear membrane, abundant endoplasmic reticulum, and much mitochondria (arrow showed). **(C)** Cells under simulated microgravity for 72 h in the SMG groups. The cell body became shrunken, and chromatin condensation and margination were observed in the cells (arrow shown). **(D)** The mitochondrial crest was broken, and the vacuolization was obvious in the SMG group (arrow showed) (**A**,**C:** 1,500×; **B**,**D:** 6,000×). CVECs, choroidal vascular endothelial cells; NG, normal gravity; SMG: simulated microgravity.

Transmission electron microscopy was employed to observe the ultrastructure of CVECs. The cells in the NG group were characterized with complete nuclear membrane, abundant mitochondria, and much endoplasmic reticulum (Figures 1A,B). After being cultured for 72 h under SMG, CVECs appeared smaller than those in the NG group. Other prominent changes discovered under the TEM included chromatin condensation and margination (Figure 1C), breakage of mitochondrial cristae, and mitochondrial vacuolation (Figure 1D).

### Immunofluorescence Staining of the F-Actin

**FIGURE 2:**
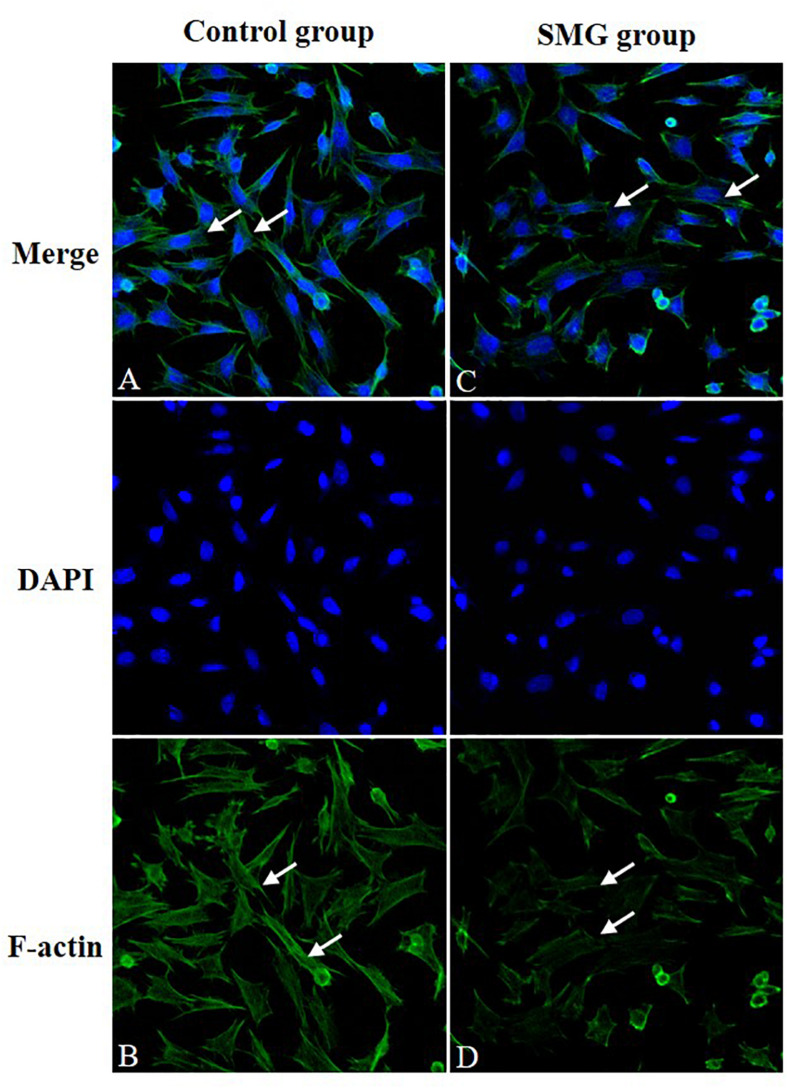
Immunofluorescence staining of F-actin. **(A,B)** CVECs cells in the normal group. The cells are characterized by strong fluorescence intensity and intact fiber structure (arrow showed). **(C,D)** CVEC cells in simulated microgravity group for 72 h. The fluorescence intensity of F-actin was weakened, and the filaments of F-actin were sparse and even slightly discontinuous (arrow showed). CVECs, choroidal vascular endothelial cells.

We performed immunofluorescence staining to determine whether there were alterations of F-actin, one of the major cell cytoskeletons, in CVECs under SMG. It showed that CVECs in the NG group exhibited high fluorescence intensity and intact fiber appearance (Figures 2A,B). In contrast, the fluorescence intensity of F-actin was low, and the filaments of F-actin were sparse and even partly discontinuous at 72 h in the SMG group (Figures 2C,D).

### The Apoptosis of CVECs

**FIGURE 3:**
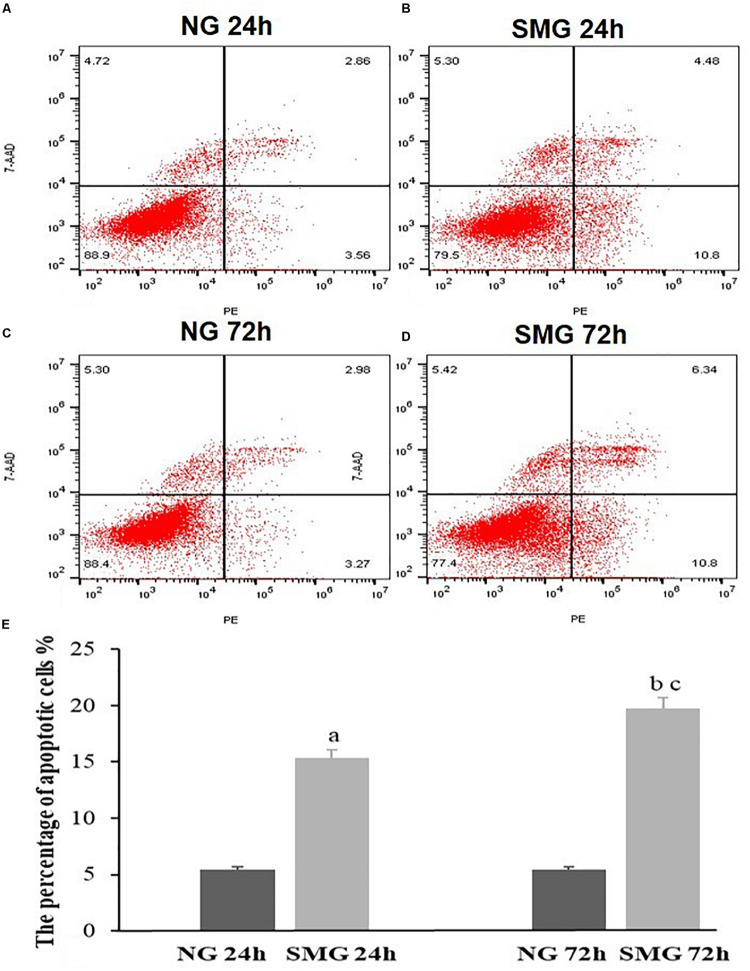
The apoptosis status of CVECs detected by Annexin V-PE/7-AAD double staining and flow cytometric analysis. Cells undergoing early apoptosis are 7AAD-/AnnexinV-PE +, while cells undergoing late apoptosis are 7AAD + /AnnexinV-PE +. The percentages of early and late apoptotic cells were summed to give the total number of apoptotic cells. **(A)** 24-h NG group, **(B)** 24-h SMG group, **(C)** 72-h NG group, **(D)** 72-h SMG group. Q1, dead cells; Q2, late apoptotic cells; Q3, normal living cells; Q4, early apoptotic cells. **(E)** Quantitative analysis of the percentage of apoptotic CVECs in the above groups. Values are presented as mean ± SD. (a) *P* < 0.001 versus 24-h NG group. (b) *P* < 0.001 versus 72-h NG group. (c) *P* < 0.001 versus 24-h SMG group (*P*-values are based on one-way analysis of variance).

We performed flow cytometry to evaluate the apoptosis rate of CVECs under SMG. The rate of apoptotic CVECs in the SMG groups at 24 and 72 h were significantly higher compared with that of the NG group (*P* < 0.001). Moreover, in the SMG group the proportion of apoptosis at 72 h was significantly higher than that at 24 h (*P* < 0.001) (Figures 3A–E).

### The mRNA Expression of Apoptosis-Related Gene in CVECs

**FIGURE 4:**
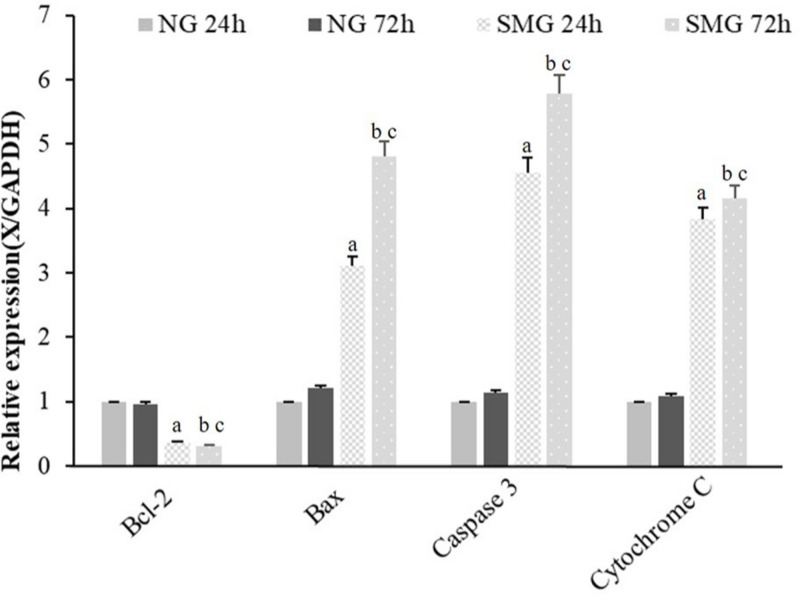
The mRNA expression of apoptosis-related gene in CVECs. Bcl-2, Bax, Caspase3, and Cytochrome C mRNA of CVECs from the NG and SMG groups were examined at 24 and 72 h, respectively, by quantitative real-time PCR. Values are presented as mean ± standard deviation. (a) *P* < 0.001 versus 24-h NG group. (b) *P* < 0.001 versus 72-h NG group. (c) *P* < 0.001 versus 24-h SMG group (*P*-values are based on one-way analysis of variance). CVECs, choroidal vascular endothelial cells.

To examine the mRNA expression of apoptosis-related genes, Bcl-2, Bax, Caspase3, and Cytochrome C mRNA of CVECs were examined at 24 and 72 h, respectively, using RT-qPCR. The relative mRNA expression levels of Bax, Caspase3, and Cytochrome C of CVECs in SMG groups at 24 and 72 h were significantly higher than those in the NG group, respectively (*P* < 0.001). Moreover, in SMG groups, the relative mRNA expression levels of these genes of CVECs at 72 h were significantly higher than those at 24 h (*P* < 0.001). The relative Bcl-2 mRNA expressions of CVECs in SMG groups at 24 and 72 h were significantly lower compared with those in the NG group, respectively (*P* < 0.001). Moreover, the relative Bcl-2 mRNA expression of CVECs at 72 h in the SMG groups was significantly lower than that at 24 h (*P* < 0.001) (Figure 4).

### The Expression of Apoptosis-Related Protein in CVECs

**FIGURE 5:**
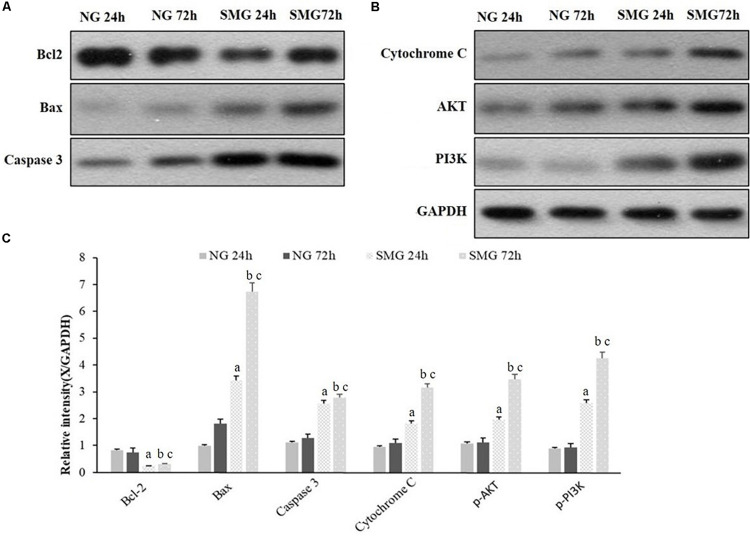
Western blot analyses of apoptosis-related protein expression in CVECs. **(A,B)** Protein expression of Bcl-2, Bax, Caspase3, Cytochrome C, p-AKT, and p-PI3K protein of CVECs were detected as a band of approximately 21, 23, 32, 12, 65, and 85 kDa, respectively, in each group. **(C)** Quantification of band intensity and statistical analysis of the relative grayscale values of these proteins in each group. Values are presented as mean ± standard deviation. (a) *P* < 0.001 versus 24-h NG group. (b) *P* < 0.001 versus 72-h NG group. (c) *P* < 0.001 versus 24-h SMG group (*P*-values are based on one-way analysis of variance).

To further explore the molecular mechanism of apoptosis under SMG in CVECs, we analyzed the protein levels of Bcl-2, Bax, Caspase3, Cytochrome C, p-AKT, and p-PI3K by Western blot. It displayed higher protein levels of Bax, Caspase3, and Cytochrome C of CVECs in the SMG groups than those in NG group at 24 and 72 h, respectively (*P* < 0.001). Moreover, in the SMG groups the protein expression at 72 h was significantly higher than that of 24 h (*P* < 0.001). In contrast, the relative Bcl-2 protein expression of CVECs in the SMG groups at 24 and 72 h were significantly lower than those in the NG group, respectively (*P* < 0.001). Moreover, in the SMG groups, Bcl-2 protein at 72 h was lower than that at 24 h (*P* < 0.001) (Figures 5A–C).

## Discussion

The study demonstrated that SMG caused ultrastructural changes in CVECs including cell shrinkage, chromatin condensation and margination, mitochondrial cavitation, and apoptotic bodies. Besides, the proportion of apoptotic CVECs under SMG increased. These observations suggested that CVECs are sensitive to SMG and SMG conditions promote CVEC apoptosis. These findings were in accordance with previous researches in many other kinds of cells under SMG (Liu et al., 2003; Zhao et al., 2016; Arun et al., 2017; Mao et al., 2018). However, there were inconsistent conclusions that no apoptosis was observed in HUVEC or dermal human microvascular endothelial cells (HMEC) cultured under SMG conditions for various times (Carlsson et al., 2003; Cotrupi and Maier, 2004; Mariotti and Maier, 2008). We suppose that the divergence is due to endothelial cell heterogeneity and different experimental conditions. Moreover, the apoptosis rate of CVECs at 72 h was higher than that at 24 h, indicating that there is a possibility of a time-dependent effect of microgravity on CVECs. This suggests that the impact of long-time spaceflight on CVECs cannot be ignored.

However, the mechanism of cell apoptosis induced by SMG is unclear. Some pro-apoptosis genes and miRNA have been found upregulated in cells under SMG which can partly elucidate the enhanced apoptosis (Boonyaratanakornkit et al., 2005; Morbidelli et al., 2005). For example, miR-503-5p which took part in inducing the apoptosis of HPMECs was overexpressed under SMG (Ferranti et al., 2014; Tang et al., 2019). However, how endothelial cells sense the SMG signals and transform them into a pro-apoptosis response remains unclear. Recent studies have proposed the cytoskeleton as a primary gravity sensor (Grimm et al., 2011; Vorselen et al., 2014). Cytoskeletal proteins are involved in cell physiology and can transmit and amplify membrane receptor-delivered signals, then alter the synthesis and secretion of cytokines, further transmit the information to the nucleus, and ultimately regulate gene expression (Geiger et al., 2001; Grimm et al., 2009, 2011; Aleshcheva et al., 2013; Ross et al., 2013). Previous studies have shown that SMG can induce prominent alterations of the F-actin cytoskeleton in many types of cells in cultivation conditions including cytoskeleton organization, cytoskeleton rearrangement, and reduction in the total amount of actin (Grosse et al., 2012; Wu et al., 2015; Corydon et al., 2016; Janmaleki et al., 2016; Costa-Almeida et al., 2018; Nassef et al., 2019). Thus, it was reasonable that cytoskeleton changes exert influence initially on signal transmitting and subsequently secretion of cytokines and gene expression, which finally lead to apoptosis.

In this study, the filaments of F-actin in SMG-exposed CVECs were found sparse and even partly discontinuous as a sign of F-actin depolymerization, demonstrating structural alteration of F-actin. Additionally, the decreased amount of F-actin was confirmed with fluorescence intensity analysis. These findings suggest that F-actin cytoskeleton alteration associated with CVEC apoptosis was induced by SMG. However the detailed mechanism remains unclear.

It is well known that harmful stress induces apoptosis in cells via the mitochondria pathway and/or Bax pathway. Bax triggers cytochrome C release from mitochondria. In contrast, Bcl-2 inhibits cytochrome C release through stabilizing the mitochondria membrane. Then, the initiator caspase is activated by the released cytochrome C. Soon after that, the effector caspase such as caspase 3 activates Dnase, ultimately resulting in DNA fragmentation. Thus Bax, cytochrome C and caspase 3 exert pro-apoptotic and Bcl-2 exerts anti-apoptotic effects in the mitochondrial pathway (Nakamura et al., 2003).

In the present study, we further examined the mitochondria pathway. It showed upregulation of Bax, cytochrome C, and caspase 3 in both gene and protein expression in CVECs under SMG. The results are consistent with the previous studies in other cell types. Many kinds of cells have demonstrated similar alterations including human osteoblastic cells (Nakamura et al., 2003), HUVECs (Li et al., 2019), human endothelial EA. hy926 cells (Infanger et al., 2007; Grimm et al., 2010), PAEC (Morbidelli et al., 2005), and human Jurkat T cells (Gasperi et al., 2014). We also found attenuated gene and protein expression of the antiapoptotic molecule Bcl-2. Similarly, some authors demonstrated a reduction of Bcl-2 gene or protein expression in endothelial cells (Morbidelli et al., 2005; Kang et al., 2011; Li et al., 2019; Tang et al., 2019), human osteoblastic cells (Nakamura et al., 2003), and carcinoma cells (Kossmehl et al., 2003). Moreover, we found that there was a time–effect relationship between the expression of Bax, cytochrome C, caspase 3, and Bcl-2 genes/proteins of CVECs and the time of SMG exposure. Thus, the gene and protein profile of the mitochondria pathway in human CVECs demonstrated that the increase of apoptosis induced by SMG was supported by the increased expression of Bax, cytochrome C, and caspase 3 and decreased expression of Bcl-2 genes and proteins.

We further examined the activated form of PI3K and Akt. The PI3K/Akt pathway regulates cell survival, proliferation, and motility. The PI3K-Akt pathway has been indicated in participating in eNOs production and inhibition of apoptosis in SMG exposed-cells (Shi et al., 2012; Ferranti et al., 2014; Arun et al., 2017; Hybel et al., 2020). In the present study, it was confirmed that the p-AKT and p-PI3K protein, phosphorylated forms of PI3K and Akt, were enhanced along with the time of exposure to SMG which indicated that the concomitant survival signal was also activated when CVEC apoptosis was triggered under SMG. However, the pathway possesses both pro-apoptotic and anti-apoptotic roles. In fact, we could not conclude whether the activated PI3K/Akt pathway is pro or anti apoptosis under SMG. Further research is needed to investigate its roles.

It is well accepted that the endothelium as a barrier lining the inner side of blood vessels orchestrates vessels and blood circulation, thus maintaining tissue homeostasis. Cell apoptosis and cytoskeleton disorganization as well as reduction would cause compromised function of choroidal endothelial cells and then might increase permeability and disrupt the integrity of vessel walls. Besides, as the cytoskeleton changes have direct influence on signal transduction, cytokine synthesis and secretion, and activated PI3K/Akt pathway regulating many cell activities, SMG appears to have potential for dysfunction of CVECs, choroidal vessels, and choroid. However, this association needs more evidence. As we know, it would be more complicated *in vivo*. Microgravity is not the only change of circumstance for the cells. Microgravity fluid shift was also believed to exert significant changes in eyes during and after long-duration spaceflight (Wostyn et al., 2019). Therefore, *in vivo* and long-term studies under stimulated microgravity and in real spaceflight should be carried out.

Taken together, our results show identified increased apoptosis; reduction and disorganization of F-actin filament; downregulation of Bcl-2 gene expression; upregulation of BAX, Caspase3, and Cytochrome C gene expression; increase in BAX, Caspase3, Cytochrome C, p-AKT, and p-PI3K protein level; and downregulation of Bcl-2 protein level in CVECs under SMG. It indicates that during the process the Bcl-2 apoptosis pathway is triggered and the PI3K/AKT pathway is concomitantly upregulated probably to counter apoptosis in this process. This study is the first to investigate the role of SMG provided by RCCS on CVECs. These altered genes and proteins reported here might provide some new insights into the mechanisms underlying microgravity-induced changes in eyes and offer opportunities to develop countermeasures.

## Data Availability Statement

The original contributions presented in the study are included in the article/supplementary material, further inquiries can be directed to the corresponding author/s.

## Author Contributions

LL was the corresponding author, carried out the concepts, design, definition of intellectual content, and reviewed manuscript. HZ and YS were co-first authors, and they both participated in the design of this study, performed the data acquisition, statistical analysis, and manuscript preparation. CQ and JZ performed statistical analysis and drafted the first manuscript. YG and CN carried out the animal experiments and collected important background information. BW, YY, and FW conducted all of the *in vitro* cell experiments. All authors have read and approved the content of the manuscript.

## Conflict of Interest

The authors declare that the research was conducted in the absence of any commercial or financial relationships that could be construed as a potential conflict of interest.
